# Do Polymorphisms Predict Hypnotherapy Response in Children With Functional Abdominal Pain Disorders: An Explorative Study

**DOI:** 10.1097/MPG.0000000000003895

**Published:** 2023-07-25

**Authors:** Clara M.A. de Bruijn, Stefan W. Hovy, Ellen Tromp, Marc A. Benninga, Kathryn T. Hall, Arine M. Vlieger

**Affiliations:** From the * Emma Children’s Hospital, Amsterdam UMC, University of Amsterdam, Pediatric Gastroenterology, Hepatology and Nutrition, Amsterdam, The Netherlands; the † Department of Pediatrics, St. Antonius Hospital, Nieuwegein, The Netherlands; the ‡ Department of Statistics, St. Antonius Hospital, Nieuwegein, The Netherlands; the § Division of Preventive Medicine, Brigham and Womens Hospital and Harvard Medical School, Boston, and Boston Public Health Commission, Boston, MA.

**Keywords:** COMT, functional abdominal pain disorders, hypnotherapy, MAO-A, OPRM1, pediatrics, polymorphisms

## Abstract

Genetic variations, in specific *COMT*, *OPRM1*, and *MAO-A* polymorphisms, have been associated with hypnotizability in adults. The aim of this exploratory study was to investigate whether these polymorphisms are also associated with response to hypnotherapy (HT) in children. Patients (8–18 years, n = 260) diagnosed with a functional abdominal pain disorder (FAPD) from a previous trial assessing HT efficacy were approached for participation and 144 agreed to collect a buccal sample. Primary aim was to explore the association between *COMT*, *OPRM1*, and *MAO-A* polymorphisms with treatment success (TS) after 3-month HT. Additionally, associations between these polymorphisms and adequate relief, anxiety, depression, quality of life, somatization, hypnotic susceptibility, expectations, pain beliefs, and coping strategies were evaluated. Participants with different variations of *COMT*, *MAO-A*, and *OPRM1* achieved similar TS levels (*P* > 0.05). No associations were found between these polymorphisms and secondary outcomes. This suggest that in pediatric patients with FAPDs, *COMT*, *OPRM1*, and *MAO-A* polymorphisms do not predict HT response.

## Graphical abstract

**Figure:**
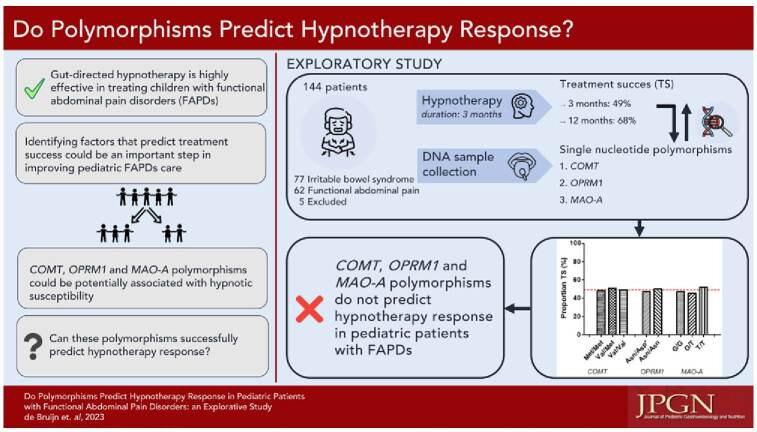

**What Is Known**

- Gut-directed hypnotherapy (HT) is an effective treatment for children with functional abdominal pain disorders (FAPDs), such as irritable bowel syndrome and functional abdominal pain syndrome.
- Hypnotic susceptibility (or hypnotizability) is shaped by suggestibility and expectancy, and have been shown to share properties with the placebo response.
- Previous studies have shown that genetic variations linked to the availability of neurotransmitters like serotonin, dopamine, and opioids are associated with the placebo response and hypnotic suggestibility in adults.

**What Is New**

- A candidate set of single nucleotide polymorphisms (*COMT*, *MAO-A*, and *OPRM1*) was not associated with HT response in pediatric patients with FAPDs.

Gut-directed hypnotherapy (HT) is highly effective in treating children with irritable bowel syndrome (IBS) or functional abdominal pain-not otherwise specified, with treatment success (TS) rates ranging from 62% to 85% (1,2). Moreover, adequate relief (AR) rates, referring to patients’ judgment of symptom improvement on an individual level and independent of symptom severity, vary from 75% to 87% (2). During HT, a state of focused awareness is induced in a patient, after which the therapist administers hypnotic suggestions to influence biological, cognitive, and emotional processes and behavior (3). An individual’s ability to experience suggested alterations in sensations, thoughts, emotions, or behavior during hypnosis is referred as hypnotic susceptibility or “hypnotizability” (3).

Underlying hypnosis mechanisms have been shown to share properties with the placebo response; both are shaped by suggestibility and expectancy (4–6). The *placebo response* is the improvement resulting from a placebo intervention: a substance or procedure without inherent power to produce any therapeutic effect (7). The placebo response includes an actual *placebo effect*, as well as other factors like, regression to the mean and spontaneous remission (8). Previous studies have shown that genetic variations linked to the availability of neurotransmitters like serotonin, dopamine, and opioids are associated with placebo responses (9–12). The genomic network underlying the placebo response is nowadays known as the “placebome” (13,14).

While most patients experience significant gain from HT, some show no or only mild symptom relief. Considering the substantial dedication required for HT, with daily listening to hypnosis exercises and regular therapist visits for a considerable period of time, identifying factors that predict TS could be an important step in improving pediatric functional abdominal pain disorders (FAPDs) care. Comparing genes in treatment responders and nonresponders may help identify underlying genetic susceptibility to HT treatment. While many genes appear to modulate placebo response, 3 single nucleotide polymorphisms (SNPs) stand out as potential candidates for modulating the response to HT in FAPDs. *COMT* and *OPRM1* are placebome SNPs that also have been associated with hypnotizability in adult patients, although results were not consistent (15–17). The third SNP, *MAO-A*, has been associated with the placebo response in adults with major depressive disorder (11). Given the high prevalence of depressive symptoms in pediatric patients with FAPDs, it may also be of interest in modulating hypnosis TS (18). The aim of this study was to investigate the moderating effect of 3 genes potentially associated with hypnotizability (*COMT*, *OPRM1*, and *MAO-A*) on the response to gut-directed HT in pediatric FAPDs.

The current exploratory study was a genetic ancillary study to an original randomized clinical trial. This FANTASIA study investigated the effectiveness of HT in pediatric FAPDs patients and was conducted between July 2011 and June 2013, enrolling 260 children aged 8–18 years with IBS or FAPs, according to the Rome III criteria (2). In the current study, only participants of the FANTASIA study that previously provided consent for future participation were eligible for participation and were contacted by phone or email (n = 235). If they agreed to participate (n = 144), a kit to collect a buccal sample was sent to their home.

The primary outcome of the current study was to determine the association of *COMT rs4680*, *OPRM1 rs1799971*, and *MAO-A rs6323* with TS after completion of 3-month HT (derived from the original FANTASIA study) (2). Secondary outcomes were associations of SNPs with TS at 12-month follow-up and AR of abdominal pain at similar time points. Additionally, associations between polymorphisms and baseline characteristics of patients were examined, including abdominal pain intensity and frequency, anxiety, depression, health-related quality of life, somatization, hypnotic susceptibility, child’s treatment expectations, pain beliefs, and coping strategies. Moreover, associations with change in abdominal pain, anxiety and depression, health-related quality of life (QoL), and somatization scores after HT treatment and at 6- and 12-month follow-up were studied. Details of the instruments used to assess these outcomes have been published previously (19).

DNA was isolated from buccal samples using Isohelix Rapidri swabs according to the manufacturer’s instructions (Cell Projects Ltd, United Kingdom). DNA sample collection occurred between January 2021 and July 2021. Genotyping and imputation of the *COMT* (rs4680), *OPRM1* (rs1799971), and *MAO-A* (rs6323) polymorphisms were performed using Infinium Screening Array (Illumina Inc, San Diego, CA) at the Human Genotyping Facility in the Erasmus MC (Rotterdam, the Netherlands). Samples with insufficient material for genetic analysis or containing too many heterozygote calls were excluded. Extended quality control containing sample call rate pruning, marker call rate pruning, Hardy-Weinberg Equilibrium determination, excess heterozygosity check, gender check, ethnic outlier check, cryptic family relatedness determination, and zCall was performed (20,21).

Differences between 2 groups were analyzed using independent *t* test for continuous variables with a symmetric distribution or Mann-Whitney *U* test for continuous variables with skewed distribution. Group differences for categorical variables were calculated using Pearson chi-squared tests. In addition, for differences between genetic polymorphisms, 1-way ANOVA for continuous variables with a symmetric distribution and Kruskal-Wallis for continuous variables with skewed distribution were used. For the analysis of the OPRM1 SNPs, subjects with the heterozygous OPRM1 genotype Asn/Asp and homozygous Asp/Asp were grouped for analysis, considering the limited number of subjects homozygous for Asp/Asp (n = 3). This is in accordance with prior research on the subject (12). Significance was set at α = 0.05. The Holm-Bonferroni correction was used to adjust for multiple testing. Statistical analyses were performed using IBM SPSS Statistics for Windows, Version 27.0 (IBM Corp., Armonk, NY). The Medical Ethics Committee of the St. Antonius Hospital, Nieuwegein, the Netherlands, approved the study and its procedures.

Of the 144 returned buccal swabs, 5 samples were excluded: too many heterozygote calls (n = 2) and genotyped gender mismatch (n = 3). All genes were in Hardy-Weinberg Equilibrium. Table 1 presents the clinical characteristics of the remaining 139 included patients and of the 121 nonparticipants. Mean age of included patients was 13.5 years (SD 2.8) and 76% were female. After correcting for multiple testing, biological sex distribution did not significantly differ between participants and nonparticipants (female: 106/139 (76%) vs 78/121 (65%), *P* = 0.037). Other baseline characteristics were not significantly different between participants and nonparticipants (Table 1, Supplemental Digital Content, http://links.lww.com/MPG/D236).

**TABLE 1.** Baseline characteristics of genotyped patients versus nonparticipating individuals from original trial

Characteristic	Genotyped patients (n = 139)	Nonparticipants (n = 121)	*P* value
Age, mean (SD), y	13.5 (2.8)	13.3 (2.8)	0.527
Female, frequency (%)	106 (76%)	78 (65%)	
Male	33 (24%)	43 (35%)	0.037
Randomization			
iHT	65 (47%)	67 (55%)	0.166
CD	74 (53%)	54 (45%)	
Diagnosis			
IBS	77 (55%)	55 (46%)	0.110
FAPS	62 (45%)	66 (54%)	
Duration of symptoms, median (IQR), y	2.3 (1.1–5.1)	2.7 (1.2–6.1)	0.427
Treatment success, frequency (%)			
3 months	N = 136; 67 (49%)	N = 101; 39 (39%)	0.103
12 months	N = 133; 90 (68%)	N = 94; 68 (72%)	0.451
Adequate relief, frequency (%)			
3 months	N = 123; 99 (81%)	N = 91; 65 (71%)	0.122
12 months	N = 133; 106 (80%)	N = 90; 76 (84%)	0.369

CD = compact disc; FAPS = functional abdominal pain syndrome; IBS = irritable bowel syndrome; IQR = interquartile range; iHT = individual hypnotherapy; SD = standard deviation.

Figure 1 shows the distribution of TS and AR rates at 3- and 12-month follow-up per genotype (*COMT rs4680*, *OPRM1 rs1799971*, *MAO-A rs6323*, respectively). Overall TS rate of included patients at 3-month was 49%. No significant differences between COMT alleles were found, with TS rates in Met/Met, Val/Met, and Val/Val genotypes of 48%, 51%, and 49%, respectively (*P* = 0.946). Differences in TS rates between *OPRM1* alleles were not significant: 50% in Asn/Asn carriers and 47% in the combined Asn/Asp and Asp/Asp group (*P* = 0.747). TS rates for *MAO-A* genotypes were: G/G: 47%, G/T: 45%, and T/T: 52% (*P* = 0.766). Overall TS of included individuals at 12-month follow-up was 68%, without significant differences between *COMT* gene polymorphisms; Met/Met: 67%, Val/Met: 65%, and Val/Val: 74% (*P* = 0.687). TS rate for *OPRM1* alleles Asn/Asn was 69%, compared to 63% in the combined Asn/Asp and Asp/Asp group (*P* = 0.564). And TS rates for MAO-A genotypes at 12-month follow-up were: G/G: 58%, G/T: 68%, and T/T: 68% (*P* = 0.768). As shown in Figure 1, differences in AR of abdominal pain at 3- and 12-month between *COMT*, *OPRM1*, and *MAO-A* alleles were not significant (*P* > 0.05). In addition, *COMT*, *OPRM1*, and *MAO-A* polymorphisms were not significantly associated with any baseline characteristics or any change in scores over time (Tables 2–8, Supplemental Digital Content, http://links.lww.com/MPG/D236).

**FIGURE 1.**
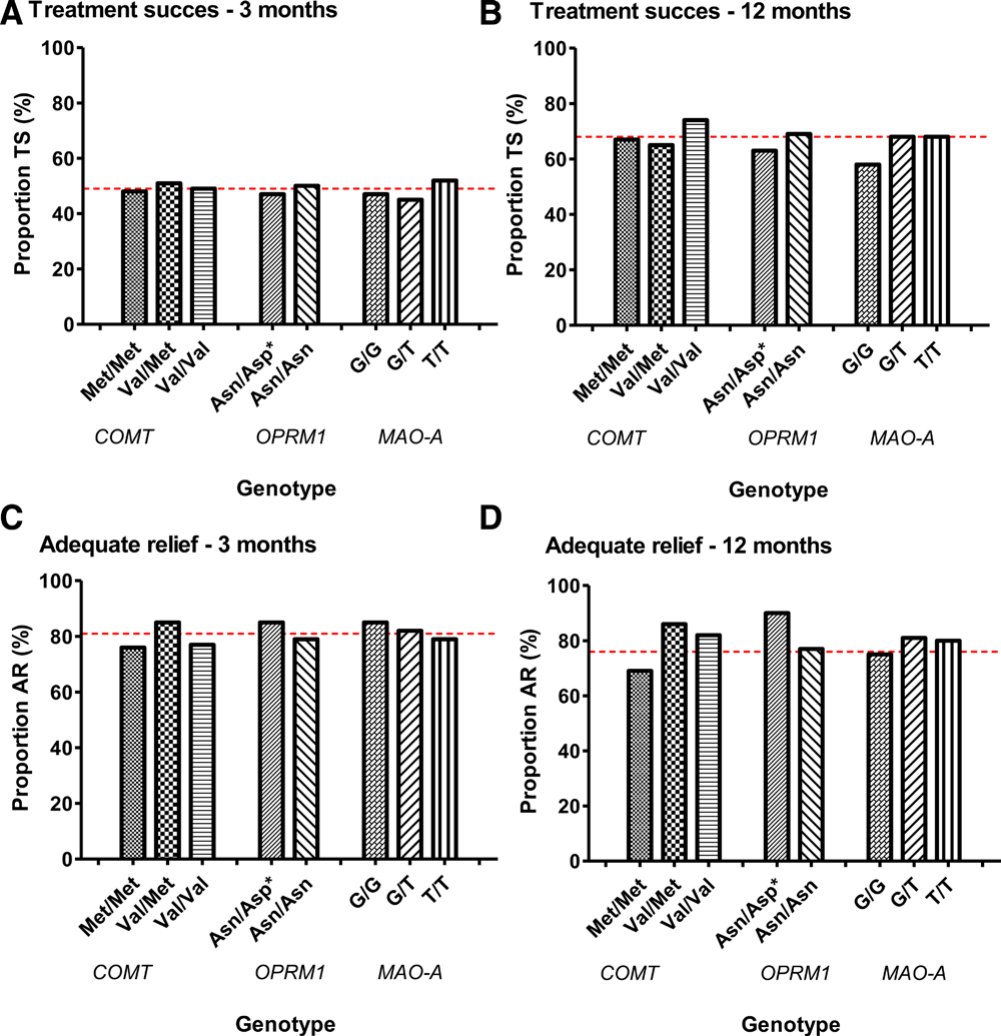
Effect of genotype on treatment success and adequate relief at 3- and 12-month follow-up. TS: treatment success was defined as at least 50% reduction in abdominal pain intensity and frequency scores compared to baseline; dotted line indicates overall treatment success of all included patients; AR: adequate relief of abdominal pain symptoms; dotted line indicates overall adequate relief of abdominal pain symptoms of all included patients; ± OPRM1 genotypes were combined for analysis (Asp/Asp or Asn/Asp vs Asn/Asn carriers). Asn = asparagine; Asp = aspartic acid; COMT = catechol-O-methyltransferase; G = guanine; Met = methionine; MAO-A = monoamine oxidase A; OPRM1 = opioid receptor mu 1; T = thymine; Val = valine

This is the first study attempting to predict response to HT in a pediatric population through genetic polymorphisms. We demonstrated that response to gut-directed HT in pediatric patients with FAPDs was not associated with *COMT*, *OPRM1*, or *MAO-A* polymorphisms. Participants in all genotype subgroups reached similar levels of TS and AR of abdominal pain after 3-month of HT and at 12-month follow-up. Moreover, baseline characteristics as described above did not differ between polymorphisms.

Contrary to results in adult patients, in which hypnotizability was associated with *COMT*, and *OPRM1* polymorphisms (15–17), our results did not show any associations between gut-directed HT response and these polymorphisms. Several reasons may explain this difference. First, it is known that hypnotizability is much higher in children compared to adults. This was confirmed in our original trial (Rutten et al, 2017), where the mean hypnotic susceptibility score of children was 6 (range 0–7). It was therefore not surprising that no association between hypnotic susceptibility and TS 12-month after the end of HT was found in the original trial (2). Interestingly though, an exploratory analysis in this ancillary study showed that all 6 participants with low hypnotizability (scores <4 out of 7) were homozygotes for Val/Val. One previous study found a similar association (15), while another had contradictory results with Val/Val carriers being more hypnotizable (17). Both studies, however, were performed in healthy adults. Our results might indicate that also in children COMT polymorphism could be a part of the complex network of factors that shape hypnotic susceptibility. Future research with adequate sample sizes, including more children with low suggestibility, is necessary to further explore this relationship. Secondly, while genetics possibly play a role in accounting for individual variations in response to HT, coping strategies and pain cognitions also mitigate abdominal pain in children and thus influence response to HT (2). Differences in coping strategies and pain cognitions between children and adults could therefore also have contributed to the conflicting results.

Till date, *MAO-A* polymorphisms have not been associated with hypnotizability or response to HT. Previous research in adult patients suffering from major depressive disorder demonstrated an association between *MAO-A* and the placebo response, but no evidence exists for their role in individuals with FAPDs (11). Given the high prevalence of depressive symptoms in patients with FAPDs, and the shared properties with the placebo response (both shaped by suggestibility and expectancy) we hypothesized that *MAO-A* might also be of interest in modulating the hypnosis response in pediatric patients (18). However, in the current study we could not demonstrate any association with *MAO-A* polymorphisms and response to gut-directed HT. It could be hypothesized that it is unlikely that a single genetic locus is fully responsible for a complex phenomenon like response to HT. Genetics influencing HT response could be regulated by a complex network of genes. Future genome-wide association studies (GWAS) powered for this analysis are necessary to further explore small genomic effects.

As mentioned previously, HT responses are likely to be regulated by a complex network of genes, with influence from many genetic loci, each with minor individual effects. This study was not adequately powered to run GWAS since it requires much larger sample sizes. Therefore, potential genetic associations may have been missed. Previous studies on this topic did find associations with even a lower number of patients. Hall et al (9) included 104 adult IBS patients and detected that the number of methionine alleles in *COMT* val158met was linearly related to the placebo response as analyzed in a regression model. Peciña et al (12) showed a correlation between the placebo effect and variations in the *OPRM1* polymorphism in only 50 subjects and, finally, the study from Presciuttini et al (16) in 43 high and 60 low hypnotizable adults demonstrated a relation between hypnotizability and *OPRM1* SNPs. With this data, we hypothesized that including at least 120 participants of the original trial would enable us to demonstrate a moderating effect on the response to gut-directed HT in pediatric FAPDs. Given the smaller number of participants in similar studies in adults, we believe the issue of being underpowered is not the main reason for the lack of finding any association. It is more likely that differences between children and adults in suggestibility have contributed to the conflicting results.

In conclusion, this study demonstrated that a candidate set of polymorphisms (*COMT*, *OPRM1*, and *MAO-A*) was not associated with response to gut-directed HT in children with FAPDs. Interestingly, we found limited evidence to support the hypothesis that the *COMT* Val/Val genotype might diminish hypnotic susceptibility. Evidence for using genetics to identify high and low HT responders in the clinical setting of pediatrics remains limited, yet it may be applicable in the future to create predictive models for personalized treatment of FAPDs.

## Acknowledgments

We acknowledge all clinicians and hypnotherapists’ great work and contributions to the original randomized clinical trial—the FANTASIA study. Finally, we thank all patients who took part in this study.

## Supplementary Material



## References

[1] Abbott RA Martin AE Newlove-Delgado TV. Psychosocial interventions for recurrent abdominal pain in childhood. Cochrane Database Syst Rev. 2017;1:CD010971. 28072460 10.1002/14651858.CD010971.pub2 PMC6464036

[2] Rutten JMTM Vlieger AM Frankenhuis C. Home-based hypnotherapy self-exercises vs individual hypnotherapy with a therapist for treatment of pediatric irritable bowel syndrome, functional abdominal pain, or functional abdominal pain syndrome: a randomized clinical trial. JAMA Pediatr. 2017;171:470–7. 28346581 10.1001/jamapediatrics.2017.0091

[3] Elkins GR Barabasz AF Council JR Spiegel D. Advancing research and practice: the revised APA Division 30 definition of hypnosis. Int J Clin Exp Hypn. 2015;63:1–9. 25365125 10.1080/00207144.2014.961870

[4] Raz A. Hypnobo: perspectives on hypnosis and placebo. Am J Clin Hypn. 2007;50:29–36. 17685242 10.1080/00029157.2007.10401595

[5] Parris BA. The prefrontal cortex and suggestion: hypnosis vs. placebo effects. Front Psychol. 2016;7:415. 27065297 10.3389/fpsyg.2016.00415 PMC4812013

[6] Parsons RD Bergmann S Wiech K Terhune DB. Direct verbal suggestibility as a predictor of placebo hypoalgesia responsiveness. Psychosom Med. 2021;83:1041–9. 34297008 10.1097/PSY.0000000000000977

[7] Stewart-Williams S Podd J. The placebo effect: dissolving the expectancy versus conditioning debate. Psychol Bull. 2004;130:324–40. 14979775 10.1037/0033-2909.130.2.324

[8] Evers AWM Colloca L Blease C. Implications of placebo and nocebo effects for clinical practice: expert consensus. Psychother Psychosom. 2018;87:204–10. 29895014 10.1159/000490354 PMC6191882

[9] Hall KT Lembo AJ Kirsch I. Catechol-O-methyltransferase val158met polymorphism predicts placebo effect in irritable bowel syndrome. PLoS One. 2012;7:e48135. 23110189 10.1371/journal.pone.0048135 PMC3479140

[10] Furmark T Appel L Henningsson S. A link between serotonin-related gene polymorphisms, amygdala activity, and placebo-induced relief from social anxiety. J Neurosci. 2008;28:13066–74. 19052197 10.1523/JNEUROSCI.2534-08.2008 PMC6671592

[11] Leuchter AF McCracken JT Hunter AM Cook IA Alpert JE. Monoamine oxidase a and catechol-o-methyltransferase functional polymorphisms and the placebo response in major depressive disorder. J Clin Psychopharmacol. 2009;29:372–7. 19593178 10.1097/JCP.0b013e3181ac4aaf

[12] Peciña M Love T Stohler CS Goldman D Zubieta J-K. Effects of the Mu opioid receptor polymorphism (OPRM1 A118G) on pain regulation, placebo effects and associated personality trait measures. Neuropsychopharmacology. 2015;40:957–65. 25308352 10.1038/npp.2014.272 PMC4330509

[13] Hall KT Loscalzo J Kaptchuk TJ. Genetics and the placebo effect: the placebome. Trends Mol Med. 2015;21:285–94. 25883069 10.1016/j.molmed.2015.02.009 PMC4573548

[14] Wang R-S Hall KT Giulianini F Passow D Kaptchuk TJ Loscalzo J. Network analysis of the genomic basis of the placebo effect. JCI insight. 2017;2:e93911. 28570268 10.1172/jci.insight.93911 PMC5453712

[15] Rominger C Weiss EM Nagl S Niederstätter H Parson W Papousek I. Carriers of the COMT Met/Met allele have higher degrees of hypnotizability, provided that they have good attentional control: a case of gene-trait interaction. Int J Clin Exp Hypn. 2014;62:455–82. 25084618 10.1080/00207144.2014.931177

[16] Presciuttini S Curcio M Sciarrino R Scatena F Jensen MP Santarcangelo EL. Polymorphism of opioid receptors μ1 in highly hypnotizable subjects. Int J Clin Exp Hypn. 2018;66:106–18. 29319460 10.1080/00207144.2018.1396128

[17] Storozheva ZI Kirenskaya AV Gordeev MN Kovaleva ME Novototsky-Vlasov VY. COMT genotype and sensory and sensorimotor gating in high and low hypnotizable subjects. Int J Clin Exp Hypn. 2018;66:83–105. 29319456 10.1080/00207144.2018.1396120

[18] Rutten JMTM Benninga MA Vlieger AM. IBS and FAPS in children: a comparison of psychological and clinical characteristics. J Pediatr Gastroenterol Nutr. 2014;59:493–9. 24897168 10.1097/MPG.0000000000000452

[19] Rutten JM Vlieger AM Frankenhuis C. Gut-directed hypnotherapy in children with irritable bowel syndrome or functional abdominal pain (syndrome): a randomized controlled trial on self exercises at home using CD versus individual therapy by qualified therapists. BMC Pediatr. 2014;14:140. 24894077 10.1186/1471-2431-14-140 PMC4060754

[20] Purcell S Neale B Todd-Brown K. PLINK: a tool set for whole-genome association and population-based linkage analyses. Am J Hum Genet. 2007;81:559–75. 17701901 10.1086/519795 PMC1950838

[21] Goldstein JI Crenshaw A Carey J. zCall: a rare variant caller for array-based genotyping: genetics and population analysis. Bioinformatics. 2012;28:2543–5. 22843986 10.1093/bioinformatics/bts479 PMC3463112

