# Supplementary material for: Do Polymorphisms Predict Hypnotherapy Response in Children With Functional Abdominal Pain Disorders: An Explorative Study
Source: J Pediatr Gastroenterol Nutr. 2023 Jul 25;77(4):486–90. doi: 10.1097/MPG.0000000000003895 (PMC10501350; doi:10.1097/MPG.0000000000003895)
Supplement: Supplementary file 1 [file mpg-77-486-s001.pdf]

	Genotyped patients (N=139)	Non-participants (N=121)	*p* - value	*Effect Size* (η^2)
**Abdominal Pain Intensity Subscale**<br>*Seven-day diary, range 0-21*	14.73<br>(4.26)<br>N=139	15.14<br>(4.63)<br>N=121	*.920*	*<.001*
**Abdominal Pain Frequency Subscale**<br>*Seven-day diary, range 0-35*	14.88<br>(5.33)<br>N=139	14.84<br>(5.67)<br>N=121	*.379*	*.003*
**Depression scores**<br>*RCADS-25, range 0-15*<br>*Mean (SD): 2.9 (2.7)*[29]	3.70<br>(2.47)<br>N=139	4.11<br>(2.38)<br>N=119	*.116*	*.010*
**Anxiety total score**<br>*RCADS-25, range 0-60*<br>*Mean (SD): 10.9 (7.8)*[29]	11.25<br>(7.81)<br>N=139	11.47<br>(8.98)<br>N=119	*.755*	*<.001*
**QoL Physical well-being**<br>*KIDSCREEN-52, [38.5- 64.3]*<br>*Mean (SD): 50.0 (10)*	45.72<br>(10.10)<br>N=139	43.05<br>(9.48)<br>N=118	*.055*	*.014*
**QoL Psychological well-being**<br>*KIDSCREEN-52, [36.9- 61.6]*<br>*Mean (SD): 50.0 (10)*	48.87<br>(9.80)<br>N=139	47.72<br>(9.88)<br>N=119	*.214*	*.006*
**QoL Moods and emotions**<br>*KIDSCREEN-52, [37.8- 62.1]*<br>*Mean (SD): 50.0 (10)*	49.71<br>(11.71)<br>N=138	47.33<br>(10.06)<br>N=119	*.097*	*.011*
**QoL Self-perception**<br>*KIDSCREEN-52, [39.2- 69.8]*<br>*Mean (SD): 50.0 (10)*	51.65<br>(10.79)<br>N=138	52.64<br>(10.02)<br>N=119	*.267*	*.005*
**QoL Autonomy**<br>*KIDSCREEN-52, [37.4- 68.8]*<br>*Mean (SD): 50.0 (10)*	51.59<br>(10.07)<br>N=139	51.64<br>(9.98)<br>N=119	*.723*	*<.001*
**QoL Relations with parents and home life**<br>*KIDSCREEN-52, [37.0- 65.9]*<br>*Mean (SD): 50.0 (10)*	55.11<br>(9.64)<br>N=139	53.28<br>(9.17)<br>N=119	*.091*	*.011*
**QoL Social support and peers**<br>*KIDSCREEN-52, [38.2- 62.7]*<br>*Mean (SD): 50.0 (10)*	49.70<br>(11.86)<br>N=139	50.25<br>(10.53)<br>N=118	*.921*	*<.001*
**QoL School environment**<br>*KIDSCREEN-52, [38.2- 61.9]*<br>*Mean (SD): 50.0 (10)*	53.30<br>(52.23)<br>N=137	51.05<br>(9.10)<br>N=117	*.112*	*.010*
**QoL Social acceptance (bullying)**<br>*KIDSCREEN-52, [35.4- 58.9]*<br>*Mean (SD): 50.0 (10)*	52.88<br>(9.55)<br>N=137	51.46<br>(9.89)<br>N=118	*.214*	*<.001*
**QoL Financial resources**<br>*KIDSCREEN-52, [37.5- 62.9]*<br>*Mean (SD): 50.0 (10)*	55.73<br>(8.70)<br>N=139	55.67<br>(9.44)<br>N=119	*.830*	*<.001*
**Children's Somatization Inventory Total score**<br>*Children's Somatization Inventory, range 0-140*<br>*Mean (SD): 10.2 (10.4)*[28]	23.91<br>(14.08)<br>N=139	26.96<br>(16.21)<br>N=119	*.141*	*<.001*
**Hypnotic susceptibility**<br>*Stanford hypnotic clinical scale for children, range 0-7*<br>*Normdata not available*	5.75<br>(1.20)<br>N=132	5.69<br>(1.20)<br>N=109	*.609*	*.001*
**Expectations; child**<br>*Treatment expectation, scale 1-10*<br>*Normdata not available*	6.99<br>(1.77)<br>N=138	6.59<br>(2.04)<br>N=118	*.161*	*.008*
**PBS Negative pain beliefs, mean (SD)**	2.17<br>(2.17)	2.25<br>(0.63)	*.262*	*.005*

Pain Beliefs Questionnaire, range 0-100<br>*Normdata not available*	N=135	N=118		
**PBS Problem focused coping potential**<br>*Pain Beliefs Questionnaire, range 0-30*<br>*Normdata not available*	1.39<br>(0.87)<br>N=139	1.33<br>(0.81)<br>N=119	*.508*	*.002*
**PBS Emotion focused coping potential**<br>*Pain Beliefs Questionnaire, range 0-30*<br>*Normdata not available*	2.32<br>(0.90)<br>N=139	2.11<br>(0.87)<br>N=119	*.069*	*.013*
**CCSC Problem focused coping, mean (SD)**<br>*Children's Coping Strategies Checklist-Revision 1, range 0-4*<br>*Normdata not available*	2.44<br>(0.54)<br>N=139	2.39<br>(0.50)<br>N=118	*.650*	*.001*
**CCSC Positive cognitive reframing**<br>*Children's Coping Strategies Checklist-Revision 1, range 0-4*<br>*Normdata not available*	2.09<br>(0.54)<br>N=139	2.13<br>(0.56)<br>N=118	*.813*	*<.001*
**CCSC Distraction strategies**<br>*Children's Coping Strategies Checklist-Revision 1, range 0-4*<br>*Normdata not available*	1.75<br>(0.37)<br>N=138	1.75<br>(0.34)<br>N=118	*.911*	*<.001*
**CCSC Avoidance strategies, mean (SD)**<br>*Children's Coping Strategies Checklist-Revision 1, range 0-4*<br>*Normdata not available*	2.14<br>(0.44)<br>N=139	2.19<br>(0.43)<br>N=119	*.213*	*.006*
**CCSC Support-seeking strategies**<br>*Children's Coping Strategies Checklist-Revision 1, range 0-4*<br>*Normdata not available*	2.13<br>(0.65)<br>N=139	2.07<br>(0.61)<br>N=118	*.549*	*.001*

eSupplement Table 1. Secondary Outcomes

Column 1 reports in consecutive order: **outcome**, *corresponding instrument and range or [10th–90th percentiles, European Normdata KIDSCREEN children and adolescents age 8-18 years],* and *normdata*.

Data are presented as mean (SD); QoL = quality of life; PBS = Pain Beliefs Questionnaire; CCSC = Children's Coping Strategies Checklist.

	Met/Met (N=43)	Val/Met (N=60)	Val/Val (N=36)	p – value	Effect Size (η^2)
**Abdominal Pain Intensity Subscale**<br>*Seven-day diary, range 0-21*	14.93<br>(3.51)<br>N=43	14.13<br>(5.00)<br>N=60	15.47<br>(3.67)<br>N=36	*.542*	*.009*
**Abdominal Pain Frequency Subscale**<br>*Seven-day diary, range 0-35*	14.95<br>(5.19)<br>N=43	13.97<br>(5.65)<br>N=60	16.31<br>(4.74)<br>N=36	*.153*	*.027*
**Depression scores**<br>*RCADS-25, range 0-15*<br>*Mean (SD): 2.9 (2.7)*[29]	3.65<br>(2.16)<br>N=43	3.63<br>(2.77)<br>N=60	3.86<br>(2.34)<br>N=36	*.739*	*.004*
**Anxiety total score**<br>*RCADS-25, range 0-60*<br>*Mean (SD): 10.9 (7.8)*[29]	12.72<br>(8.94)<br>N=43	10.92<br>(7.11)<br>N=60	10.06<br>(7.43)<br>N=36	*.294*	*.018*
**QoL Physical well-being**<br>*KIDSCREEN-52, [38.5- 64.3]*<br>*Mean (SD): 50.0 (10)*	47.29<br>(11.17)<br>N=43	45.97<br>(10.11)<br>N=60	43.42<br>(8.45)<br>N=36	*.259*	*.020*
**QoL Psychological well-being**<br>*KIDSCREEN-52, [36.9- 61.6]*<br>*Mean (SD): 50.0 (10)*	49.51<br>(10.32)<br>N=43	48.01<br>(9.22)<br>N=60	49.53<br>(10.27)<br>N=36	*.799*	*.003*
**QoL Moods and emotions**<br>*KIDSCREEN-52, [37.8- 62.1]*<br>*Mean (SD): 50.0 (10)*	49.85<br>(12.72)<br>N=43	48.03<br>(10.87)<br>N=59	52.30<br>(11.64)<br>N=36	*.292*	*.018*
**QoL Self-perception**<br>*KIDSCREEN-52, [39.2- 69.8]*<br>*Mean (SD): 50.0 (10)*	52.45<br>(11.93)<br>N=43	51.09<br>(10.37)<br>N=59	51.59<br>(10.27)<br>N=36	*.918*	*.001*
**QoL Autonomy**<br>*KIDSCREEN-52, [37.4- 68.8]*<br>*Mean (SD): 50.0 (10)*	51.95<br>(9.98)<br>N=43	51.00<br>(10.24)<br>N=60	52.14<br>(10.14)<br>N=36	*.608*	*.007*
**QoL Relations with parents and home life**<br>*KIDSCREEN-52, [37.0- 65.9]*<br>*Mean (SD): 50.0 (10)*	55.55<br>(10.44)<br>N=43	53.82<br>(9.81)<br>N=60	56.76<br>(8.21)<br>N=36	*.385*	*.014*
**QoL Social support and peers**<br>*KIDSCREEN-52, [38.2- 62.7]*<br>*Mean (SD): 50.0 (10)*	48.04<br>(13.60)<br>N=43	49.19<br>(10.63)<br>N=60	52.54<br>(11.39)<br>N=36	*.385*	*.014*
**QoL School environment**<br>*KIDSCREEN-52, [38.2- 61.9]*<br>*Mean (SD): 50.0 (10)*	55.02<br>(10.74)<br>N=42	52.20<br>(8.48)<br>N=59	53.07<br>(10.11)<br>N=36	*.272*	*.019*
**QoL Social acceptance (bullying)**<br>*KIDSCREEN-52, [35.4- 58.9]*<br>*Mean (SD): 50.0 (10)*	52.73<br>(9.64)<br>N=42	51.90<br>(10.25)<br>N=59	54.67<br>(8.14)<br>N=36	*.418*	*.013*
**QoL Financial resources**<br>*KIDSCREEN-52, [37.5- 62.9]*<br>*Mean (SD): 50.0 (10)*	55.19<br>(8.87)<br>N=43	54.63<br>(9.61)<br>N=60	58.19<br>(6.28)<br>N=36	*.206*	*.023*
**Children's Somatization Inventory Total score**<br>*Children's Somatization Inventory, range 0-140*<br>*Mean (SD): 10.2 (10.4)*[28]	22.58<br>(13.29)<br>N=43	24.18<br>(15.15)<br>N=60	25.03<br>(13.36)<br>N=36	*.735*	*.004*
**Hypnotic susceptibility**<br>*Stanford hypnotic clinical scale for children, range 0-7*<br>*Normdata not available*	5.83<br>(0.93)<br>N=42	5.93<br>(1.03)<br>N=57	5.35<br>(1.65)<br>N=33	*.352*	*.016*
**Expectations; child**<br>*Treatment expectation, scale 1-10*<br>*Normdata not available*	7.12<br>(1.99)<br>N=43	7.04<br>(1.53)<br>N=59	6.75<br>(1.90)<br>N=36	*.550*	*.009*
**PBS Negative pain beliefs**	2.18<br>(0.60)	2.07<br>(0.64)	2.31<br>(0.59)	*.440*	*.012*

Pain Beliefs Questionnaire, range 0-100<br>*Normdata not available*	N=43	N=58	N=34		
**PBS Problem focused coping potential**<br>*Pain Beliefs Questionnaire, range 0-30*<br>*Normdata not available*	1.36<br>(0.78)<br>N=43	1.49<br>(0.97)<br>N=60	1.27<br>(0.80)<br>N=36	*.574*	*.008*
**PBS Emotion focused coping potential**<br>*Pain Beliefs Questionnaire, range 0-30*<br>*Normdata not available*	2.26<br>(0.78)<br>N=43	2.36<br>(1.01)<br>N=60	2.31<br>(0.84)<br>N=36	*.822*	*.003*
**CCSC Problem focused coping**<br>*Children's Coping Strategies Checklist-Revision 1, range 0-4*<br>*Normdata not available*	2.35<br>(0.60)<br>N=43	2.44<br>(0.49)<br>N=60	2.55<br>(0.54)<br>N=36	*.202*	*.023*
**CCSC Positive cognitive reframing**<br>*Children's Coping Strategies Checklist-Revision 1, range 0-4*<br>*Normdata not available*	2.09<br>(0.62)<br>N=43	2.04<br>(0.49)<br>N=60	2.19<br>(0.53)<br>N=36	*.318*	*.017*
**CCSC Distraction strategies**<br>*Children's Coping Strategies Checklist-Revision 1, range 0-4*<br>*Normdata not available*	1.70<br>(0.46)<br>N=43	1.78<br>(0.33)<br>N=59	1.76<br>(0.34)<br>N=36	*.326*	*.016*
**CCSC Avoidance strategies**<br>*Children's Coping Strategies Checklist-Revision 1, range 0-4*<br>*Normdata not available*	2.15<br>(0.40)<br>N=43	2.16<br>(0.50)<br>N=60	2.10<br>(0.40)<br>N=36	*.918*	*.001*
**CCSC Support-seeking strategies**<br>*Children's Coping Strategies Checklist-Revision 1, range 0-4*<br>*Normdata not available*	2.14<br>(0.68)<br>N=43	2.03<br>(0.59)<br>N=60	2.27<br>(0.71)<br>N=36	*.242*	*.021*

eSupplement Table 2. Differences in secondary outcomes at baseline by *COMT* alleles

Column 1 reports in consecutive order: **outcome**, *corresponding instrument and range or [10th–90th percentiles, European Normdata KIDSCREEN children and adolescents age 8-18 years],* and *normdata*.

Data are presented as mean (SD); QoL = quality of life; PBS = Pain Beliefs Questionnaire; CCSC = Children's Coping Strategies Checklist.

	Asn/Asn (N=109)	Asn/Asp (N=27) and Asp/Asp (N= 3)	*p* – value	*Effect Size* (η^2)
**Abdominal Pain Intensity Subscale**<br>*Seven-day diary, range 0-21*	14.75<br>(4.30)<br>N=109	14.63<br>(4.17)<br>N=30	*.975*	*<.001*
**Abdominal Pain Frequency Subscale**<br>*Seven-day diary, range 0-35*	14.92<br>(5.37)<br>N=109	14.73<br>(5.29)<br>N=30	*.841*	*<.001*
**Depression scores**<br>*RCADS-25, range 0-15*<br>*Mean (SD): 2.9 (2.7)*[29]	3.83<br>(2.48)<br>N=109	3.23<br>(2.40)<br>N=30	*.201*	*.012*
**Anxiety total score**<br>*RCADS-25, range 0-60*<br>*Mean (SD): 10.9 (7.8)*[29]	11.82<br>(8.11)<br>N=109	9.20<br>(6.29)<br>N=30	*.105*	*.019*
**QoL Physical well-being**<br>*KIDSCREEN-52, [38.5- 64.3]*<br>*Mean (SD): 50.0 (10)*	45.48<br>(10.41)<br>N=109	46.58<br>(8.98)<br>N=30	*.517*	*.003*
**QoL Psychological well-being**<br>*KIDSCREEN-52, [36.9- 61.6]*<br>*Mean (SD): 50.0 (10)*	48.43<br>(9.96)<br>N=109	50.44<br>(9.19)<br>N=30	*.309*	*.008*
**QoL Moods and emotions**<br>*KIDSCREEN-52, [37.8- 62.1]*<br>*Mean (SD): 50.0 (10)*	48.81<br>(11.33)<br>N=108	52.95<br>(12.67)<br>N=30	*.150*	*.015*
**QoL Self-perception**<br>*KIDSCREEN-52, [39.2- 69.8]*<br>*Mean (SD): 50.0 (10)*	51.48<br>(10.52)<br>N=108	52.25<br>(11.89)<br>N=30	*.959*	*<.001*
**QoL Autonomy**<br>*KIDSCREEN-52, [37.4- 68.8]*<br>*Mean (SD): 50.0 (10)*	51.83<br>(10.31)<br>N=109	50.69<br>(9.29)<br>N=30	*.396*	*.005*
**QoL Relations with parents and home life**<br>*KIDSCREEN-52, [37.0- 65.9]*<br>*Mean (SD): 50.0 (10)*	55.21<br>(9.58)<br>N=109	54.76<br>(10.00)<br>N=30	*.775*	*.001*
**QoL Social support and peers**<br>*KIDSCREEN-52, [38.2- 62.7]*<br>*Mean (SD): 50.0 (10)*	49.32<br>(12.33)<br>N=109	51.06<br>(10.03)<br>N=30	*.841*	*<.001*
**QoL School environment**<br>*KIDSCREEN-52, [38.2- 61.9]*<br>*Mean (SD): 50.0 (10)*	53.71<br>(9.83)<br>n=107	51.82<br>(9.00)<br>N=30	*.352*	*.006*
**QoL Social acceptance (bullying)**<br>*KIDSCREEN-52, [35.4- 58.9]*<br>*Mean (SD): 50.0 (10)*	52.64<br>(9.74)<br>n=107	53.74<br>(8.93)<br>N=30	*.624*	*.025*
**QoL Financial resources**<br>*KIDSCREEN-52, [37.5- 62.9]*<br>*Mean (SD): 50.0 (10)*	56.08<br>(8.09)<br>N=109	54.45<br>(10.70)<br>N=30	*.657*	*.001*
**Children's Somatization Inventory Total score**<br>*Children's Somatization Inventory, range 0-140*<br>*Mean (SD): 10.2 (10.4)*[28]	24.91<br>(14.13)<br>N=109	20.27<br>(13.51)<br>N=30	*.065*	*.025*
**Hypnotic susceptibility**<br>*Stanford hypnotic clinical scale for children, range 0-7*<br>*Normdata not available*	5.74<br>(1.19)<br>n=104	5.82<br>(1.28)<br>n=28	*.658*	*.001*
**Expectations; child**<br>*Treatment expectation, scale 1-10*<br>*Normdata not available*	7.04<br>(1.75)<br>n=108	6.8<br>(1.88)<br>N=30	*.664*	*.001*
**PBS Negative pain beliefs**	2.21<br>(0.60)	2.01<br>(0.66)	*.185*	*.013*

Pain Beliefs Questionnaire, range 0-100<br>*Normdata not available*	n=106	n=29		
**PBS Problem focused coping potential**<br>*Pain Beliefs Questionnaire, range 0-30*<br>*Normdata not available*	1.37<br>(0.87)<br>N=109	1.47<br>(0.89)<br>N=30	*.683*	*.001*
**PBS Emotion focused coping potential**<br>*Pain Beliefs Questionnaire, range 0-30*<br>*Normdata not available*	2.29<br>(0.92)<br>N=109	2.42<br>(0.79)<br>N=30	*.668*	*.001*
**CCSC Problem focused coping**<br>*Children's Coping Strategies Checklist-Revision 1, range 0-4*<br>*Normdata not available*	2.44<br>(0.54)<br>N=109	2.41<br>(0.54)<br>N=30	*.992*	*<.001*
**CCSC Positive cognitive reframing**<br>*Children's Coping Strategies Checklist-Revision 1, range 0-4*<br>*Normdata not available*	2.11<br>(0.54)<br>N=109	2.04<br>(0.56)<br>N=30	*.396*	*.005*
**CCSC Distraction strategies**<br>*Children's Coping Strategies Checklist-Revision 1, range 0-4*<br>*Normdata not available*	1.80<br>(0.37)<br>n=108	1.58<br>(0.34)<br>N=30	*.007*	*.053*
**CCSC Avoidance strategies**<br>*Children's Coping Strategies Checklist-Revision 1, range 0-4*<br>*Normdata not available*	2.17<br>(0.46)<br>N=109	2.03<br>(0.38)<br>N=30	*.168*	*.014*
**CCSC Support-seeking strategies**<br>*Children's Coping Strategies Checklist-Revision 1, range 0-4*<br>*Normdata not available*	2.13<br>(0.65)<br>N=109	2.11<br>(0.66)<br>N=30	*.747*	*.001*

eSupplement Table 3. Differences in secondary outcomes at baseline by *OPRM1* alleles

Column 1 reports in consecutive order: **outcome**, *corresponding instrument and range or [10th–90th percentiles, European Normdata KIDSCREEN children and adolescents age 8-18 years],* and *normdata*.

Data are presented as mean (SD); QoL = quality of life; PBS = Pain Beliefs Questionnaire; CCSC = Children's Coping Strategies Checklist.

	G/G (N=15)	G/T (N=42)	T/T (N=82)	*p* -value	*Effect Size* (η^2)
**Abdominal Pain Intensity Subscale**<br>*Seven-day diary, range 0-21*	13.27<br>(4.95)<br>N=15	15.17<br>(3.38)<br>N=42	14.77<br>(4.51)<br>N=82	*.537*	*.009*
**Abdominal Pain Frequency Subscale**<br>*Seven-day diary, range 0-35*	14.27<br>(6.02)<br>N=15	15.67<br>(4.76)<br>N=42	14.59<br>(5.50)<br>N=82	*.595*	*.008*
**Depression scores**<br>*RCADS-25, range 0-15*<br>*Mean (SD): 2.9 (2.7)*[29]	4.07<br>(2.79)<br>N=15	4.14<br>(2.23)<br>N=42	3.40<br>(2.51)<br>N=82	*.117*	*.031*
**Anxiety total score**<br>*RCADS-25, range 0-60*<br>*Mean (SD): 10.9 (7.8)*[29]	9.67<br>(7.29)<br>N=15	13.05<br>(9.28)<br>N=42	10.62<br>(6.97)<br>N=82	*.302*	*.017*
**QoL Physical well-being**<br>*KIDSCREEN-52, [38.5- 64.3]*<br>*Mean (SD): 50.0 (10)*	50.80<br>(14.85)<br>N=15	43.13<br>(6.91)<br>N=42	46.11<br>(10.15)<br>N=82	*.069*	*.039*
**QoL Psychological well-being**<br>*KIDSCREEN-52, [36.9- 61.6]*<br>*Mean (SD): 50.0 (10)*	50.19<br>(12.12)<br>N=15	47.61<br>(8.35)<br>N=42	49.27<br>(10.08)<br>N=82	*.543*	*.009*
**QoL Moods and emotions**<br>*KIDSCREEN-52, [37.8- 62.1]*<br>*Mean (SD): 50.0 (10)*	50.00<br>(14.22)<br>N=15	46.24<br>(10.94)<br>N=42	51.45<br>(11.35)<br>N=81	*.063*	*.040*
**QoL Self-perception**<br>*KIDSCREEN-52, [39.2- 69.8]*<br>*Mean (SD): 50.0 (10)*	54.39<br>(12.81)<br>N=15	49.20<br>(9.96)<br>N=42	52.40<br>(10.71)<br>N=81	*.197*	*.024*
**QoL Autonomy**<br>*KIDSCREEN-52, [37.4- 68.8]*<br>*Mean (SD): 50.0 (10)*	52.81<br>(14.10)<br>N=15	51.16<br>(9.69)<br>N=42	51.60<br>(9.52)<br>N=82	*.779*	*.004*
**QoL Relations with parents and home life**<br>*KIDSCREEN-52, [37.0- 65.9]*<br>*Mean (SD): 50.0 (10)*	54.68<br>(8.73)<br>N=15	54.44<br>(9.93)<br>N=42	55.54<br>(9.73)<br>N=82	*.771*	*.004*
**QoL Social support and peers**<br>*KIDSCREEN-52, [38.2- 62.7]*<br>*Mean (SD): 50.0 (10)*	47.48<br>(15.54)<br>N=15	49.01<br>(12.26)<br>N=42	50.46<br>(10.95)<br>N=82	*.765*	*.004*
**QoL School environment**<br>*KIDSCREEN-52, [38.2- 61.9]*<br>*Mean (SD): 50.0 (10)*	53.82<br>(12.73)<br>N=15	52.37<br>(10.01)<br>N=41	53.67<br>(8.90)<br>N=81	*.306*	*.017*
**QoL Social acceptance (bullying)**<br>*KIDSCREEN-52, [35.4- 58.9]*<br>*Mean (SD): 50.0 (10)*	56.11<br>(7.23)<br>N=15	52.68<br>(9.79)<br>N=41	52.39<br>(9.78)<br>N=81	*.292*	*.018*
**QoL Financial resources**<br>*KIDSCREEN-52, [37.5- 62.9]*<br>*Mean (SD): 50.0 (10)*	57.33<br>(8.96)<br>N=15	56.47<br>(9.03)<br>N=42	55.05<br>(8.51)<br>N=82	*.335*	*.016*
**Children's Somatization Inventory Total score**<br>*Children's Somatization Inventory, range 0-140*<br>*Mean (SD): 10.2 (10.4)*[28]	21.20<br>(11.18)<br>N=15	27.93<br>(15.96)<br>N=42	22.34<br>(13.23)<br>N=82	*.138*	*.029*
**Hypnotic susceptibility**<br>*Stanford hypnotic clinical scale for children, range 0-7*<br>*Normdata not available*	6.27<br>(0.67)<br>N=13	5.91<br>(1.16)<br>N=42	5.58<br>(1.27)<br>N=77	*.120*	*.032*
**Expectations; child**<br>*Treatment expectation, scale 1-10*<br>*Normdata not available*	6.73<br>(1.67)<br>N=15	6.63<br>(1.85)<br>N=41	7.21<br>(1.74)<br>N=82	*.196*	*.024*
**PBS Negative pain beliefs**	2.01<br>(0.69)	2.27<br>(0.61)	2.14<br>(0.61)	*.379*	*.014*

Pain Beliefs Questionnaire, range 0-100<br>*Normdata not available*	N=15	N=40	N=80		
**PBS Problem focused coping potential**<br>*Pain Beliefs Questionnaire, range 0-30*<br>*Normdata not available*	1.56<br>(0.91)<br>N=15	1.41<br>(0.70)<br>N=42	1.35<br>(0.95)<br>n=82	*.665*	*.006*
**PBS Emotion focused coping potential**<br>*Pain Beliefs Questionnaire, range 0-30*<br>*Normdata not available*	2.57<br>(0.97)<br>N=15	2.31<br>(0.83)<br>N=42	2.28<br>(0.92)<br>N=82	*.422*	*.013*
**CCSC Problem focused coping**<br>*Children's Coping Strategies Checklist-Revision 1, range 0-4*<br>*Normdata not available*	2.28<br>(0.53)<br>N=15	2.42<br>(0.48)<br>N=42	2.47<br>(0.57)<br>N=82	*.413*	*.013*
**CCSC Positive cognitive reframing**<br>*Children's Coping Strategies Checklist-Revision 1, range 0-4*<br>*Normdata not available*	2.01<br>(0.61)<br>N=15	2.13<br>(0.46)<br>N=42	2.09<br>(0.57)<br>N=82	*.900*	*.002*
**CCSC Distraction strategies**<br>*Children's Coping Strategies Checklist-Revision 1, range 0-4*<br>*Normdata not available*	1.68<br>(0.31)<br>N=15	1.79<br>(0.35)<br>N=42	1.74<br>(0.40)<br>N=81	*.611*	*.007*
**CCSC Avoidance strategies**<br>*Children's Coping Strategies Checklist-Revision 1, range 0-4*<br>*Normdata not available*	2.17<br>(0.35)<br>N=15	2.19<br>(0.38)<br>N=42	2.11<br>(0.49)<br>N=82	*.493*	*.010*
**CCSC Support-seeking strategies**<br>*Children's Coping Strategies Checklist-Revision 1, range 0-4*<br>*Normdata not available*	1.96<br>(0.54)<br>N=15	2.21<br>(0.74)<br>N=42	2.11<br>(0.62)<br>N=82	*.624*	*.007*

eSupplement Table 4. Differences in secondary outcomes at baseline by *MAO-A* alleles

Column 1 reports in consecutive order: **outcome**, *corresponding instrument and range or [10th–90th percentiles, European Normdata KIDSCREEN children and adolescents age 8-18 years],* and *normdata*.

Data are presented as mean (SD); QoL = quality of life; PBS = Pain Beliefs Questionnaire; CCSC = Children's Coping Strategies Checklist.

	COMT			*OPRM1*		*MAO-A*		
	**Met/Met (N=43)**	**Val/Met (N=60)**	**Val/Val (N=36)**	**Asn/Asn (N=109)**	**Asn/Asp (N=27) and Asp/Asp (N= 3)**	**G/G (N=15)**	**G/T (N=42**	**T/T (N=82)**
**Abdominal Pain Intensity Subscale**<br>*Seven-day diary, range 0-21*	-6.95<br>(6.33)<br>N=42	-7.03<br>(6.86)<br>N=59	-7.86<br>(6.88)<br>N=35	-7.03<br>(6.90)<br>N=106	-7.63<br>(5.62)<br>N=30	-6.40<br>(6.91)<br>N=15	-6.95<br>(5.64)<br>N=42	-7.52<br>(7.17)<br>N=79
**Abdominal Pain Frequency Subscale**<br>*Seven-day diary, range 0-35*	-7.26<br>(6.35)<br>N=42	-7.61<br>(7.04)<br>N=59	-8.14<br>(7.41)<br>N=35	-7.48<br>(7.18)<br>N=106	-8.23<br>(5.56)<br>N=30	-6.47<br>(9.16)<br>N=15	-7.76<br>(5.16)<br>N=42	-7.80<br>(7.27)<br>N=79
**Depression scores**<br>*RCADS-25, range 0-15*<br>*Mean (SD): 2.9 (2.7)*[29]	-0.38<br>(2.19)<br>N=41	-0.32<br>(1.84)<br>N=59	-0.50<br>(1.75)<br>N=34	-0.43<br>(1.99)<br>N=104	-0.28<br>(1.69)<br>N=23	-0.57<br>(1.60)<br>N=14	-0.26<br>(2.30)<br>N=42	-0.41<br>(1.76)<br>N=78
**Anxiety total score**<br>*RCADS-25, range 0-60*<br>*Mean (SD): 10.9 (7.8)*[29]	-1.83<br>(5.04)<br>N=41	-0.93<br>(5.48)<br>N=59	0.68<br>(5.88)<br>N=34	-1.31<br>(5.77)<br>N=104	0.35<br>(4.45)<br>N=30	0.50<br>(5.53)<br>N=14	-0.79<br>(5.86)<br>N=42	-1.04<br>(5.33)<br>N=78
**QoL Physical well-being**<br>*KIDSCREEN-52, [38.5- 64.3]*<br>*Mean (SD): 50.0 (10)*	2.66<br>(9.54)<br>N=40	2.72<br>(8.64)<br>N=58	2.87<br>(8.51)<br>N=34	2.88<br>(8.78)<br>N=103	4.63<br>(9.54)<br>N=29	0.64<br>(11.28)<br>N=14	3.44<br>(8.43)<br>N=41	2.98<br>(8.53)<br>N=77
**QoL Psychological well-being**<br>*KIDSCREEN-52, [36.9- 61.6]*<br>*Mean (SD): 50.0 (10)*	2.10<br>(9.21)<br>N=41	4.15<br>(9.30)<br>N=59	2.06<br>(10.04)<br>N=34	2.22<br>(8.88)<br>N=104	6.17<br>(9.41)<br>N=30	-0.95<br>(9.07)<br>N=14	2.68<br>(9.09)<br>N=42	3.87<br>(9.63)<br>N=78
**QoL Moods and emotions**<br>*KIDSCREEN-52, [37.8- 62.1]*<br>*Mean (SD): 50.0 (10)*	2.50<br>(10.61)<br>N=40	4.90<br>(8.51)<br>N=58	-1.76<br>(9.70)<br>N=34	3.12<br>(9.75)<br>N=102	1.03<br>(9.31)<br>N=30	0.54<br>(8.68)<br>N=14	3.34<br>(9.20)<br>N=42	2.32<br>(10.36)<br>N=76
**QoL Self-perception**<br>*KIDSCREEN-52, [39.2- 69.8]*<br>*Mean (SD): 50.0 (10)*	0.15<br>(10.34)<br>N=41	2.02<br>(8.03)<br>N=58	-0.93<br>(4.67)<br>N=34	0.29<br>(8.22)<br>N=103	1.83<br>(7.82)<br>N=30	-0.70<br>(5.02)<br>N=14	-0.12<br>(7.13)<br>N=42	1.38<br>(9.16)<br>N=77

**QoL Autonomy**<br>*KIDSCREEN-52, [37.4- 68.8]*<br>*Mean (SD): 50.0 (10)*	3.42<br>(10.66)<br>N=41	1.90<br>(10.73)<br>N=59	0.13<br>(8.56)<br>N=34	1.13<br>(9.95)<br>N=104	3.95<br>(9.48)<br>N=30	2.65<br>(11.55)<br>N=14	0.96<br>(11.10)<br>N=42	2.29<br>(9.54)<br>N=78
**QoL Relations with parents and home life**<br>*KIDSCREEN-52, [37.0- 65.9]*<br>*Mean (SD): 50.0 (10)*	-1.14<br>(7.96)<br>N=41	0.84<br>(8.01)<br>N=59	-0.26<br>(7.56)<br>N=34	-0.12<br>(8.19)<br>N=104	0.01<br>(6.69)<br>N=30	0.81<br>(4.75)<br>N=14	-1.11<br>(9.05)<br>N=42	0.38<br>(7.66)<br>N=78
**QoL Social support and peers**<br>*KIDSCREEN-52, [38.2- 62.7]*<br>*Mean (SD): 50.0 (10)*	3.66<br>(14.42)<br>N=41	1.28<br>(11.97)<br>N=59	-0.90<br>(9.61)<br>N=34	1.29<br>(12.73)<br>N=104	1.96<br>(10.74)<br>N=30	2.54<br>(12.46)<br>N=14	2.17<br>(13.22)<br>N=42	0.87<br>(11.85)<br>N=78
**QoL School environment**<br>*KIDSCREEN-52, [38.2- 61.9]*<br>*Mean (SD): 50.0 (10)*	0.78<br>(11.37)<br>N=39	1.41<br>(8.42)<br>N=55	0.75<br>(11.43)<br>N=34	1.02<br>(8.52)<br>N=100	3.58<br>(9.01)<br>N=28	2.58<br>(9.62)<br>N=14	-2.07<br>(11.01)<br>N=40	2.43<br>(9.49)<br>N=74
**QoL Social acceptance (bullying)**<br>*KIDSCREEN-52, [35.4- 58.9]*<br>*Mean (SD): 50.0 (10)*	0.70<br>(8.51)<br>N=40	2.82<br>(8.51)<br>N=58	-0.21<br>(8.65)<br>N=34	1.96<br>(8.29)<br>N=102	-1.44<br>(8.48)<br>N=30	0.21<br>(7.18)<br>N=14	1.65<br>(8.54)<br>N=41	1.48<br>(8.91)<br>N=77
**QoL Financial resources**<br>*KIDSCREEN-52, [37.5- 62.9]*<br>*Mean (SD): 50.0 (10)*	1.47<br>(8.19)<br>N=41	1.78<br>(8.59)<br>N=59	-1.69<br>(9.24)<br>N=34	0.38<br>(7.22)<br>N=104	2.64<br>(10.00)<br>N=30	2.68<br>(8.78)<br>N=14	-1.34<br>(9.98)<br>N=42	1.62<br>(7.80)<br>N=80
**Children's Somatization Inventory Total score**<br>*Children's Somatization Inventory, range 0-140*<br>*Mean (SD): 10.2 (10.4)*[28]	-5.39<br>(9.39)<br>N=41	-6.14<br>(11.01)<br>N=59	-3.62<br>(11.83)<br>N=34	-5.90<br>(11.09)<br>N=104	-3.79<br>(9.63)<br>N=30	-0.79<br>(7.21)<br>N=14	-5.36<br>(9.16)<br>N=42	-6.03<br>(11.89)<br>N=78

eSupplement Table 5. Change in secondary outcomes after 3-month follow-up

Column 1 reports in consecutive order: **outcome**, *corresponding instrument and range or [10th–90th percentiles, European Normdata KIDSCREEN children and adolescents age 8-18 years]*, and *normdata*. Data are presented as mean (SD); QoL = quality of life; PBS = Pain Beliefs Questionnaire; CCSC = Children's Coping Strategies Checklist.

	COMT			*OPRM1*		*MAO-A*		
	**Met/Met (N=43)**	**Val/Met (N=60)**	**Val/Val (N=36)**	**Asn/Asn (N=109)**	**Asn/Asp (N=27) and Asp/Asp (N= 3)**	**G/G (N=15)**	**G/T (N=42)**	**T/T (N=82)**
**Abdominal Pain Intensity Subscale**<br>*Seven-day diary, range 0-21*	-7.37<br>(6.37)<br>N=41	-8.59<br>(6.58)<br>N=58	-7.49<br>(7.75)<br>N=35	-8.35<br>(6.73)<br>N=104	-7.20<br>(7.09)<br>N=30	-6.85<br>(6.23)<br>N=13	-7.60<br>(6.38)<br>N=42	-8.28<br>(7.18)<br>N=79
**Abdominal Pain Frequency Subscale**<br>*Seven-day diary, range 0-35*	-8.10<br>(6.92)<br>N=41	-8.55<br>(7.42)<br>N=58	-7.43<br>(7.88)<br>N=35	-8.55<br>(7.43)<br>N=104	-7.23<br>(7.21)<br>N=30	-6.85<br>(7.01)<br>N=13	-8.21<br>(6.59)<br>N=42	-8.28<br>(7.85)<br>N=79
**Depression scores**<br>*RCADS-25, range 0-15*<br>*Mean (SD): 2.9 (2.7)*[29]	-0.20<br>(2.16)<br>N=40	-0.45<br>(2.33)<br>N=58	-1.06<br>(2.01)<br>N=35	-0.69<br>(2.22)<br>N=104	-0.03<br>(2.11)<br>N=29	-0.54<br>(2.57)<br>N=13	-0.66<br>(2.24)<br>N=41	-0.47<br>(2.15)<br>N=79
**Anxiety total score**<br>*RCADS-25, range 0-60*<br>*Mean (SD): 10.9 (7.8)*[29]	-2.45<br>(5.99)<br>N=40	-1.45<br>(5.56)<br>N=58	-0.11<br>(5.02)<br>N=35	-1.81<br>(5.88)<br>N=104	0.07<br>(3.95)<br>N=29	1.08<br>(6.34)<br>N=13	-1.17<br>(5.65)<br>N=41	-1.92<br>(5.38)<br>N=79
**QoL Physical well-being**<br>*KIDSCREEN-52, [38.5- 64.3]*<br>*Mean (SD): 50.0 (10)*	0.09<br>(9.28)<br>N=36	4.57<br>(10.73)<br>N=57	2.30<br>(10.48)<br>N=32	3.50<br>(10.80)<br>N=97	1.98<br>(8.57)<br>N=28	-2.56<br>(12.44)<br>N=13	1.90<br>(10.28)<br>N=37	4.00<br>(9.82)<br>N=75
**QoL Psychological well-being**<br>*KIDSCREEN-52, [36.9- 61.6]*<br>*Mean (SD): 50.0 (10)*	2.88<br>(9.71)<br>N=40	4.02<br>(9.77)<br>N=58	2.09<br>(9.71)<br>N=35	3.31<br>(9.00)<br>N=104	2.10<br>(11.16)<br>N=29	-4.01<br>(8.06)<br>N=13	2.38<br>(9.15)<br>N=41	4.76<br>(9.73)<br>N=79
**QoL Moods and emotions**<br>*KIDSCREEN-52, [37.8- 62.1]*<br>*Mean (SD): 50.0 (10)*	3.49<br>(8.02)<br>N=40	7.92<br>(9.82)<br>N=57	1.32<br>(9.59)<br>N=35	5.43<br>(9.75)<br>N=103	3.20<br>(9.51)<br>N=29	0.99<br>(7.44)<br>N=13	5.30<br>(9.21)<br>N=41	5.22<br>(10.08)<br>N=78
**QoL Self-perception**<br>*KIDSCREEN-52, [39.2- 69.8]*<br>*Mean (SD): 50.0 (10)*	-1.85<br>(7.84)<br>N=40	0.63<br>(9.79)<br>N=57	-0.98<br>(8.97)<br>N=35	-1.53<br>(8.57)<br>N=103	1.66<br>(8.45)<br>N=29	-3.63<br>(8.85)<br>N=13	-0.60<br>(8.87)<br>N=41	-0.07<br>(9.16)<br>N=78

**QoL Autonomy**<br>*KIDSCREEN-52, [37.4- 68.8]*<br>*Mean (SD): 50.0 (10)*	0.04<br>(9.91)<br>N=39	2.75<br>(9.57)<br>N=58	-0.54<br>(12.29)<br>N=35	0.83<br>(10.31)<br>N=103	1.32<br>(10.63)<br>N=29	0.18<br>(13.96)<br>N=12	0.23<br>(11.55)<br>N=41	1.66<br>(9.36)<br>N=79
**QoL Relations with parents and home life**<br>*KIDSCREEN-52, [37.0- 65.9]*<br>*Mean (SD): 50.0 (10)*	-2.77<br>(7.19)<br>N=39	0.93<br>(7.52)<br>N=58	-0.09<br>(9.46)<br>N=35	0.08<br>(8.13)<br>N=103	-1.55<br>(7.77)<br>N=29	-3.90<br>(8.64)<br>N=12	-0.16<br>(7.82)<br>N=41	-0.05<br>(8.11)<br>N=79
**QoL Social support and peers**<br>*KIDSCREEN-52, [38.2- 62.7]*<br>*Mean (SD): 50.0 (10)*	2.92<br>(13.99)<br>N=39	2.02<br>(10.24)<br>N=58	-0.01<br>(9.14)<br>N=35	2.37<br>(11.22)<br>N=103	0.03<br>(11.29)<br>N=29	-1.06<br>(10.75)<br>N=12	2.51<br>(12.79)<br>N=41	1.78<br>(10.42)<br>N=79
**QoL School environment**<br>*KIDSCREEN-52, [38.2- 61.9]*<br>*Mean (SD): 50.0 (10)*	-2.07<br>(9.89)<br>N=37	4.11<br>(9.90)<br>N=52	-0.21<br>(9.03)<br>N=35	0.44<br>(10.10)<br>N=96	1.16<br>(8.84)<br>N=28	-1.72<br>(9.21)<br>N=12	0.97<br>(9.87)<br>N=39	1.55<br>(10.16)<br>N=73
**QoL Social acceptance (bullying)**<br>*KIDSCREEN-52, [35.4- 58.9]*<br>*Mean (SD): 50.0 (10)*	-0.21<br>(5.85)<br>N=39	3.54<br>(11.05)<br>N=54	0.63<br>(10.48)<br>N=35	2.04<br>(9.28)<br>N=99	-0.00<br>(10.29)<br>N=29	-0.25<br>(10.03)<br>N=13	0.29<br>(8.30)<br>N=39	2.59<br>(10.23)<br>N=76
**QoL Financial resources**<br>*KIDSCREEN-52, [37.5- 62.9]*<br>*Mean (SD): 50.0 (10)*	-0.60<br>(10.72)<br>N=39	2.48<br>(10.95)<br>N=57	-1.66<br>(6.17)<br>N=34	0.38<br>(9.20)<br>N=102	1.36<br>(12.76)<br>N=28	2.19<br>(7.12)<br>N=13	-2.24<br>(11.50)<br>N=41	1.64<br>(9.25)<br>N=76
**Children's Somatization Inventory Total score**<br>*Children's Somatization Inventory, range 0-140*<br>*Mean (SD): 10.2 (10.4)*[28]	-4.93<br>(10.68)<br>N=40	-8.91<br>(11.91)<br>N=57	-6.71<br>(12.94)<br>N=35	-7.83<br>(12.23)<br>N=103	-5.10<br>(11.10)<br>N=29	-2.46<br>(9.12)<br>N=13	-6.73<br>(13.35)<br>N=40	-8.09<br>(11.41)<br>N=79

eSupplement Table 6. Change in secondary outcomes after 6-month follow-up

Column 1 reports in consecutive order: **outcome**, *corresponding instrument and range or [10th–90th percentiles, European Normdata KIDSCREEN children and adolescents age 8-18 years]*, and *normdata*. Data are presented as mean (SD); QoL = quality of life; PBS = Pain Beliefs Questionnaire; CCSC = Children's Coping Strategies Checklist.

	COMT			*OPRM1*		*MAO-A*		
	**Met/Met (N=43)**	**Val/Met (N=60)**	**Val/Val (N=36)**	**Asn/Asn (N=109)**	**Asn/Asp (N=27) and Asp/Asp (N= 3)**	**G/G (N=15)**	**G/T (N=42)**	**T/T (N=82)**
**Abdominal Pain Intensity Subscale** <u>*Seven-day diary, range 0-21*</u>	-9.36 (6.71) N=42	-9.32 (6.75) N=57	-10.15 (6.66) N=34	-9.54 (6.96) N=103	-9.63 (5.54) N=30	-7.92 (7.29) N=12	-9.44 (6.58) N=41	-9.84 (6.67) N=80
**Abdominal Pain Frequency Subscale** <u>*Seven-day diary, range 0-35*</u>	-9.62 (7.82) N=42	-9.44 (7.06) N=57	-9.82 (7.40) N=34	-9.74 (7.61) N=103	-9.43 (6.52) N=30	-9.25 (6.80) N=12	-9.46 (7.27) N=41	-9.71 (7.54) N=80
**Depression scores** <u>*RCADS-25, range 0-15*</u> *Mean (SD): 2.9 (2.7)*[29]	-0.57 (2.26) N=42	-0.81 (2.17) N=57	-1.00 (2.19) N=34	-0.77 (2.23) N=103	-0.86 (2.20) N=30	-1.41 (1.73) N=12	-0.61 (2.25) N=41	-0.78 (2.23) N=80
**Anxiety total score** <u>*RCADS-25, range 0-60*</u> *Mean (SD): 10.9 (7.8)*[29]	-3.07 (6.35) N=42	-2.07 (5.99) N=57	-1.21 (5.24) N=34	-2.33 (6.20) N=103	-1.31 (5.04) N=30	-3.92 (6.23) N=12	-2.15 (6.17) N=41	-1.91 (5.78) N=80
**QoL Physical well-being** <u>*KIDSCREEN-52, [38.5- 64.3]*</u> *Mean (SD): 50.0 (10)*	1.60 (12.05) N=40	5.22 (10.85) N=56	2.42 (10.04) N=33	3.35 (10.66) N=99	5.18 (12.90) N=30	1.40 (9.63) N=12	3.60 (10.86) N=38	3.57 (11.48) N=79
**QoL Psychological well-being** <u>*KIDSCREEN-52, [36.9- 61.6]*</u> *Mean (SD): 50.0 (10)*	4.99 (11.52) N=42	5.09 (9.58) N=56	2.51 (11.95) N=33	3.97 (11.13) N=101	5.30 (10.07) N=30	1.88 (11.78) N=12	4.77 (11.44) N=40	4.61 (10.44) N=79
**QoL Moods and emotions** <u>*KIDSCREEN-52, [37.8- 62.1]*</u> *Mean (SD): 50.0 (10)*	5.17 (10.85) N=42	7.44 (10.79) N=56	0.99 (11.44) N=33	6.19 (11.56) N=101	2.35 (8.99) N=30	5.52 (10.91) N=12	5.30 (10.91) N=12	4.91 (11.70) N=79
**QoL Self-perception** <u>*KIDSCREEN-52, [39.2- 69.8]*</u> *Mean (SD): 50.0 (10)*	-2.92 (9.69) N=42	1.56 (9.11) N=56	-1.72 (6.54) N=33	-1.47 (8.43) N=101	1.35 (10.57) N=30	-1.28 (5.74) N=12	-0.30 (8.04) N=40	-0.82 (9.75) N=79

**QoL Autonomy**<br>*KIDSCREEN-52, [37.4- 68.8]*<br>*Mean (SD): 50.0 (10)*	1.25<br>(11.06)<br>N=42	3.96<br>(10.33)<br>n=57	1.14<br>(13.22)<br>N=33	1.71<br>(12.01)<br>N=102	5.24<br>(8.78)<br>N=30	2.92<br>(11.83)<br>N=12	1.80<br>(12.83)<br>N=40	2.61<br>(10.59)<br>N=80
**QoL Relations with parents and home life**<br>*KIDSCREEN-52, [37.0- 65.9]*<br>*Mean (SD): 50.0 (10)*	-2.06<br>(8.29)<br>N=42	1.51<br>(8.31)<br>N=57	-0.34<br>(9.19)<br>N=33	-0.91<br>(8.42)<br>N=102	3.72<br>(7.94)<br>N=30	-0.44<br>(7.46)<br>N=12	0.16<br>(8.47)<br>N=40	-0.17<br>(8.92)<br>N=80
**QoL Social support and peers**<br>*KIDSCREEN-52, [38.2- 62.7]*<br>*Mean (SD): 50.0 (10)*	6.55<br>(13.56)<br>N=42	3.44<br>(10.25)<br>N=57	1.71<br>(12.01)<br>N=33	4.54<br>(12.55)<br>N=102	3.36<br>(10.08)<br>N=30	8.00<br>(16.81)<br>N=12	3.52<br>(13.11)<br>N=40	3.63<br>(10.36)<br>N=80
**QoL School environment**<br>*KIDSCREEN-52, [38.2- 61.9]*<br>*Mean (SD): 50.0 (10)*	1.65<br>(9.64)<br>N=40	3.10<br>(9.11)<br>N=55	-1.17<br>(10.58)<br>N=33	0.66<br>(9.81)<br>N=98	3.15<br>(8.82)<br>N=30	-1.46<br>(9.44)<br>N=12	1.81<br>(9.01)<br>N=39	1.89<br>(10.18)<br>N=77
**QoL Social acceptance (bullying)**<br>*KIDSCREEN-52, [35.4- 58.9]*<br>*Mean (SD): 50.0 (10)*	2.02<br>(8.55)<br>N=41	2.03<br>(10.63)<br>N=54	-0.17<br>(9.27)<br>N=33	1.57<br>(10.17)<br>N=98	0.83<br>(8.12)<br>N=30	-3.42<br>(10.54)<br>N=12	0.47<br>(9.90)<br>N=38	2.76<br>(9.16)<br>N=78
**QoL Financial resources**<br>*KIDSCREEN-52, [37.5- 62.9]*<br>*Mean (SD): 50.0 (10)*	2.19<br>(9.49)<br>N=42	2.08<br>(10.34)<br>N=55	-0.68<br>(6.00)<br>N=33	1.08<br>(8.26)<br>N=100	2.55<br>(11.86)<br>N=30	3.02<br>(9.38)<br>N=12	1.47<br>(10.63)<br>N=40	1.14<br>(8.37)<br>N=78
**Children's Somatization Inventory Total score**<br>*Children's Somatization Inventory, range 0-140*<br>*Mean (SD): 10.2 (10.4)*[28]	-4.90<br>(10.60)<br>N=42	-8.82<br>(11.25)<br>N=57	-8.32<br>(13.25)<br>N=34	-7.97<br>(12.40)<br>N=103	-5.97<br>(9.68)<br>N=30	-4.67<br>(8.21)<br>N=12	-8.05<br>(11.52)<br>N=41	-7.58<br>(12.18)<br>N=80

eSupplement Table 7. Change in secondary outcomes after 12-month follow-up

Column 1 reports in consecutive order: **outcome**, *corresponding instrument and range or [10th–90th percentiles, European Normdata KIDSCREEN children and adolescents age 8-18 years]*, and *normdata*. Data are presented as mean (SD); QoL = quality of life; PBS = Pain Beliefs Questionnaire; CCSC = Children's Coping Strategies Checklist.
